# A Biodegradable Polyurethane Dermal Matrix in Reconstruction of Free Flap Donor Sites: A Pilot Study

**Published:** 2015-04-24

**Authors:** Marcus J.D. Wagstaff, Bradley J. Schmitt, Patrick Coghlan, James P. Finkemeyer, Yugesh Caplash, John E. Greenwood

**Affiliations:** ^a^Adult Burn Service, Royal Adelaide Hospital, North Terrace, Adelaide, South Australia, Australia; ^b^Department of Plastic and Reconstructive Surgery, Royal Adelaide Hospital, North Terrace, Adelaide, South Australia, Australia

**Keywords:** biodegradable polyurethane, synthetic dermal matrix, dermal scaffold, free flap donor site, reconstruction

## Abstract

We have developed a biodegradable temporizing matrix (BTM) capable of supporting secondary split-skin graft-take in animal studies. We report its first long-term implantation and use as a dermal scaffold in humans. This preliminary study assesses its ability to integrate, its ease of delamination, its ability to sustain split-skin graft in complex wounds, the degree of wound contraction, and ultimately the quality of the scar at 1 year postimplantation. Ten patients were recruited, each requiring elective free flap reconstruction. Free flap donor sites created were anterolateral thigh flaps, fibular osseocutaneous flaps, or radial/ulnar forearm (RF/UF) flaps. The BTM was implanted when the flap was detached from its donor site. Dressing changes were performed twice weekly. The time elapsed between implantation and delamination depended on the type of flap and thus the wound bed left. Once integrated, the BTMs were delaminated in theatre, and the surface of the “neodermis” was refreshed by dermabrasion, prior to application of a split-skin graft. The BTM integration occurred in all patients (100% in 6 patients, with 90%, 84%, 76%, and 60% integration in the remainder). Integrated BTM sustained successful graft-take in all patients. Complete take was marred in 2 patients, over areas of BTM that had not integrated and graft application was performed too early. The BTM can be applied into wounds in humans and can integrate, persist in the presence of infection, and sustain split-skin overgrafting, despite the trial group presenting with significant comorbidities.

We have been developing a totally synthetic dermal replacement scaffold for use in major burn injury, using a polyurethane open-cell foam (Novosorb).^1^^-^7 The primary aims of this dressing are (1) to temporize extensive debrided wounds while waiting for donor site recovery for delayed split-skin grafting, (2) to allow integration of vascular tissue into the foam to create a neodermis, (3) to sustain split-skin grafting once integrated, and (4) to reduce wound contraction during the remodeling phase. The secondary goal is to provide a platform onto which an autologous cultured composite skin can be applied, thus eliminating the need for extensive split-skin graft harvest.

The foam is 2-mm-thick and biodegradable by hydrolysis, with a nonbiodegradable polyurethane seal bonded to the upper surface. Unlike other dermal replacement technologies, it does not contain any biological molecules such as collagen, potentially offering a greater resistance to infection. This has been reported in porcine studies with up to 4 weeks’ exposure and stable graft take. The bonded seal prevents evaporative water loss and, after integration, is peeled away to expose the vascularized foam for skin grafting.

Successful integration and split-skin graft take has been demonstrated in controlled wounds in pigs over 4 weeks and the design was further optimized. To assess the safety of short-term human implantation, we performed a randomized control trial, using the unsealed foam in a thick form as a topical negative pressure in pressure ulcers, with the foam replaced every 2 to 3 days over an 8-week period. No adverse reactions were noted, and the foam was performed as effectively as the control dressing.^8^

To study long-term implantation, integration, contraction, and scar outcomes, we proposed its use in a cohort of elective, complex, full-thickness wounds normally requiring skin graft cover, namely free-flap donor sites. In particular, split-skin graft cover over the radial forearm flap^9^ donor site often results in graft breakdown over the flexor carpi radialis, subsequently requiring debridement of the tendon to allow healing by secondary intention.^10^^-^16 This can lead to scar tethering and reduced power during wrist flexion. The use of other dermal substitutes has been reported in such wounds to improve outcomes.^17^^-^20

This study therefore reports on the first human long-term implantation of the biodegradable temporizing matrix (BTM) in 10 patients with free-flap donor sites. Quantitative, qualitative, and photographic data have been collected from wound creation, implantation, through the integration phase to split-skin grafting and through to scar outcomes at 1 year postimplantation. Our reflection on the handling and outcomes of this study has led to further optimization of the BTM for use in a subsequent cohort of 10 patients.

## OBJECTIVES OF THIS STUDY

Objectives were to (*a*) report on any apparent local and systemic response to the presence of the BTM, (*b*) qualitatively assess the ease of delamination of the bonded seal and the BTM's ability in complex wounds to integrate and sustain split-skin graft, and (*c*) assess the progression of scar contracture and ultimate quality of the scar at 1-year postimplantation.

## MATERIALS AND METHODS

The study was approved by the Royal Adelaide Hospital Human Research Ethics Committee (HREC110613), authorized by the Therapeutic Goods Administration (CTN104/2011) and registered with the Australian and New Zealand Clinical Trials Registry (ACTRN12611000753954).

Ten patients were recruited, each needing surgery necessitating the harvest of an anterolateral thigh (ALT) flap (3), a fibular osseocutaneous (FOC) flap (3), or a radial/ulnar forearm (RF/UF) flap (4).

The Biodegradable Temporising Matrix (BTM) was manufactured, packaged, and sterilized by γ-irradiation by PolyNovo Biomaterials Pty Ltd, Port Melbourne, Victoria, Australia.

Once the free flap was detached, the BTM was unpacked, cut to wound shape and applied, seal uppermost, to the donor site. Its margin was secured with surgical staples. In ALT and FOC sites, a wound drain was inserted into deep potential spaces.

The BTM was overdressed with Mepitel (Mölnlycke, Gothenburg, Sweden) and Acticoat (Smith & Nephew, Hull, UK) held with Hypafix (BSN Medical, Hamburg, Germany). Compression was afforded by crêpe bandage. RF/UF and FOC sites were splinted for 7 days to limit shearing forces due to underlying tendon movement. Subsequent similar dressing changes and wound assessments were performed every 3 to 4 days (twice weekly) by the investigators. Prior to BTM application, and at dressing changes, clinical photographs were taken and the wound circumference was traced onto Visitrak acetates (Smith & Nephew, Hull, UK), from which the wound area was calculated using a Visitrak reader.

On the basis of porcine studies,^5^^,^^6^ material delamination and grafting were scheduled to be performed around day 21 postimplantation. Integration was subjectively assessed at the time of skin grafting by peeling an edge of the seal back to view a test area of the underlying polyurethane matrix. The appearance of fine granulations at the superficial surface combined with obscuring of the foam structure indicated integration with neovascularization. For cases that had been partially delaminated prior to graft surgery, integration could be directly visualized during dressing changes. As the trial progressed, for reasons discussed later, the day of grafting became variable.

At the time of split-skin grafting, the BTM seal was removed (delamination) in theatre by peeling. The qualitative ease of delamination was noted. The surface of the “neodermis” was refreshed by light dermabrasion prior to split-skin graft application. Areas of nonadherent/nonintegrated or excised BTM were measured using a Visitrak tracing as previously described and expressed as a percentage of the total wound area. With the exception of patient 2 (which was meshed), all grafts were hand fenestrated with a scalpel. Grafts were affixed with running 6/0 poliglecaprone sutures (Monocryl, Ethicon Inc, Somerville, NJ), or surgical staples. Initial graft dressings were with Jelonet petrolatum gauze (Smith & Nephew, Hull, UK) and povidone-iodine (Betadine, Sanofi, Paris, France)–soaked gauze, secured with crêpe bandages. The first dressing change and graft check were done at day 5, with subsequent dressing changes every 3 to 4 days. Mobilization commenced in the graft area at day 5 postapplication. Graft take was defined as percentage wound surface area covered by adherent, vascularized skin after 7 days postapplication by Visitrak measurement.

Patients with unhealed areas underwent continued dressing management as clinically indicated, typically with Acticoat and Hypafix until healed. Patients with healed grafts were advised to moisturize their grafts twice daily throughout the 1-year trial period with an aqueous-based moisturizer, Sorbolene (Redwin, New South Wales, Australia). Further Visitrak measurements were taken at episodes determined by the patient attendance to outpatient reviews for a further year.

Scar appearance was assessed using 2 scar assessment scales at approximately 1 year postimplantation by a single physiotherapist. The scar assessment scales used were The Patient and Observer Scar Assessment Scale v2.0 (POSAS)^21^ and Matching Assessment using Photographs with Scars (MAPS).^22^

The POSAS consists of both a Patient and Observer scale. The Patient scale of the POSAS consists of 6 questions on a scale of 1 to 10 with a score of 1, representing the best scar or sensation and a score of 10 representing the worst. The Observer scale of the POSAS consists of 6 questions on scar characteristics on a scale of 1 to 10, lower scores indicating a better result.

For each scale, results therefore can range from 6 to 60. An additional question for overall opinion of the scar (score 1-10) is included in both scales but not included in the overall score. It has been reported to have superior internal consistency and reliability than the Vancouver scar scale,^23^^,^^24^ recommended for use where only 1 measure of the scar is required and to gain the patient's perspective of their scar.^25^

The MAPS is a scar assessment scale rating 4 scar parameters (surface, border height, thickness, and color) on a 6-point scale from −1 to 4 with the option of including pigmentation as required. Possible scores range from −5 to 17, with a score approaching 0 representing a better outcome. Although not used widely, MAPS has been shown to have acceptable levels of intra- and interrater reliability.^22^^,^^26^

The patient first completed the POSAS Patient Scale, which was then followed by the observer completing the MAPS and POSAS Observer Scale.

## RESULTS

A temporal pictorial record of each patient's course was generated, a selection of which has been included. Table 1 contains a summary of the outcomes of each case, including wound surface areas expressed as a percentage of the original wound at the time of definitive skin grafting and at the nearest time point to death (patients 1 and 2) or 1 year postimplantation. Scar scores at 1 year are also included. Additional pertinent information for each case is presented later.

### Patient 1

The BTM appeared to integrate despite the patient's obvious physical frailty and slowness to heal in his other wounds. In theatre on day 22, following delamination, we saw that part of the polymer had failed to adhere and integrate over the intermuscular septum, due to the presence of an encapsulated seroma. This portion of the BTM was excised (24% of the total). The remainder was dermabraded and the skin graft applied. Despite an apparently vascularized bed, including over the muscle where the BTM had been removed, the graft failed except for a crescentic fragment along the lateral margin (over the integrated BTM), covering 14.5% of the total wound area.

The failed graft was removed on the ward and the wound redressed. The integrated BTM was refreshed by dermabrasion and the residual wound regrafted on day 34. Residual BTM was visibly retained in the wound following the second dermabrasion. He was discharged with 100% graft take. Shortly after an assessment at day 71, he died secondary to pneumonia. No scar assessment was made.

### Patient 2

The BTM was inspected on day 3 and the patient appeared healthy. On day 6, the BTM showed gray discoloration along its medial border and on day 10, infection was clinically obvious. Incontinence secondary to a urinary tract infection had resulted in infected urine leaking onto his wound dressing. *Enterobacter* was cultured. The central part of the BTM (37% of the total) was excised. The wound underwent daily cleaning and fresh antimicrobial dressings (Acticoat). The remaining BTM (63%) continued to integrate into the wound. Delamination of the residual seal, surface dermabrasion, and split-skin grafting were performed at day 20. The graft was meshed to reduce potential graft problems related to the previous infection. There was 100% skin graft take across the whole wound. His last assessment was at day 213 prior to his death due to metastatic disease. No scar assessment was made.

### Patient 3

The BTM was delaminated at day 20 and had failed to adhere and integrate over the peroneus longus tendon. This portion was removed (10% of the total wound area). The skin graft took anteriorly over the integrated BTM but failed posteriorly over the exposed tendon. Further grafting was unnecessary.

### Patient 4

**Figure 1:**
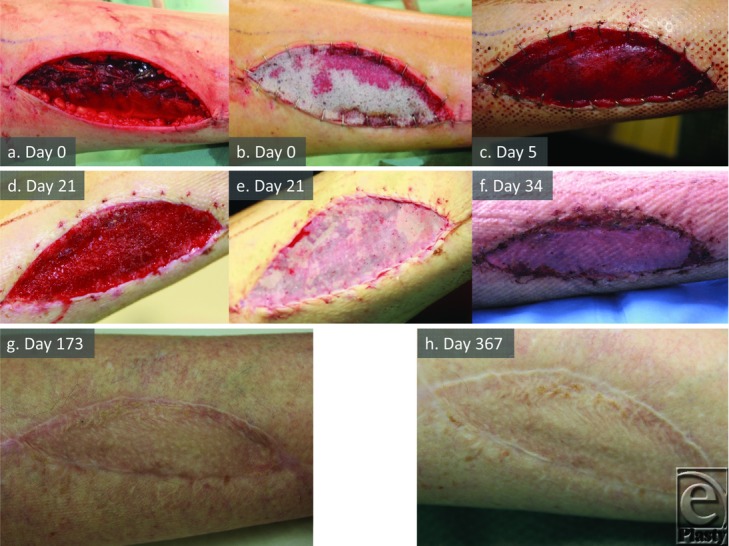
Patient 4 temporal series (FOC). Implantation (a,b), integration (c,d), grafting (e), graft take (f), and maturation (g,h) to 1 year.

The clinical course proceeded uneventfully (Fig 1).

### Patient 5

The BTM integrated fully; however, delamination, dermabrasion, and split-skin grafting were delayed until day 29 due to issues relating to the tumour site reconstruction.

### Patient 6 (Fig 2)

A serous collection under the central area was noted (day 17) (overlying the tendon sheaths) with delayed BTM integration compared to peripheral areas. The seal was partially removed on day 19 allowing fluid escape. The BTM dermal component integrated over the next week. At day 36, the residual seal was delaminated, the BTM dermabraded, and split-skin graft was applied, which took completely.

### Patient 7

The BTM integrated without issue. Readmitted for delamination, dermabrasion, and successful split-skin grafting at day 34 (the over-tendon integration time set by experience with patient 6).

### Patient 8

**Figure 3:**
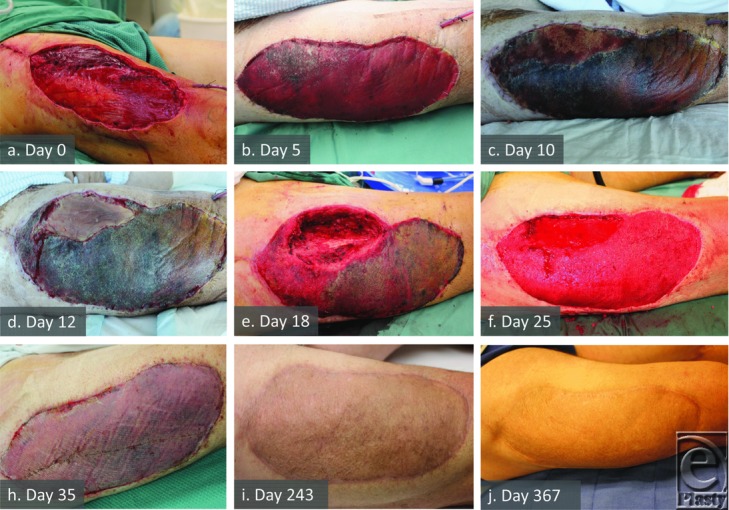
Anterolateral thigh flap donor site at creation (a), note the muscular color difference in proximomedial quadrant. Biodegradable temporizing matrix (BTM) integration (b) marred in the same quadrant. Obvious muscle nonviability (c,d) followed by excision of the nonviable rectus femoris and release of a coliform/anaerobe abscess deep to the dead muscle leaving defect (e). Note that the remainder of the BTM is intact despite the abscess proximity although delaminated in the proximolateral quadrant. After the negative pressure wound therapy (NPWT) treatment, total BTM delamination and dermabrasion (f). Graft 10 days later (h) and with maturation to 12 months (i,j).

During flap harvest, part of the rectus femoris muscle was devitalized (Fig 3). The BTM overlying this compromised muscle failed to adhere and was removed on day 10 (representing 16% of the total BTM on Visitrak measurement). On day 18, the muscle was obviously necrotic and required surgical debridement. A coliform/anaerobe abscess was discovered under the necrotic muscle. Its proximity had not affected the integration of the remaining adjacent BTM. A topical negative pressure dressing was applied to the whole wound. At day 25, the dressing was removed and the remaining 84% of the BTM was delaminated, dermabraded, and grafted, with 100% graft take across the whole wound.

### Patient 9

**Figure 4:**
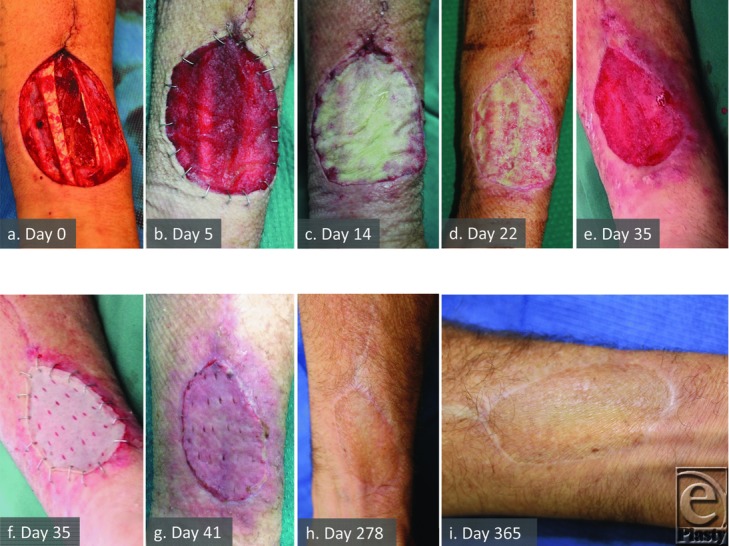
Patient 9 temporal series (RF) illustrating infection and resolution. Complex wound bed at flap harvest (a), progression of integration (b), and infection within the matrix at 14 days (c). Treatment by partial delamination and topical wound management eradicates the infection while the matrix persists (d). By day 35, BTM is integrated and grafted (e,f). Graft take (g), remodeling, and maturation (h,i).

On day 14, the BTM seal lifted over a turbid fluid collection (Fig 4). This area of raised seal was excised and a fluid sample sent for microbiological culture. *Staphylococcus aureus* was reported. Intravenous antibiotics were commenced and antimicrobial dressings were changed daily. The BTM persisted and subsequently fully integrated. The patient demonstrated no outward sign or symptom related to this infection. He was readmitted at day 35 for delamination of the residual seal, dermabrasion, and split-skin grafting.

### Patient 10

On day 19, a turbid collection was noted under the BTM seal, lifting the seal locally. He was asymptomatic. The seal was windowed to allow fluid escape. A daily antimicrobial dressing regime was commenced with oral antibiotic administration. A wound fluid sample cultured mixed anaerobes. The BTM integration appeared slow over the tendons, and skin grafting was scheduled for 6 weeks postimplantation. However, domestic issues postponed delamination of the residual seal, dermabrasion, and skin grafting to day 49 (7 weeks). Although the skin graft adhered at 4 days postapplication and had visibly taken and was maturing at day 61, the central graft over the flexor carpi radialis tendon broke down following gardening trauma first noted at day 106. The underlying tendon was not exposed and remained covered with vascularized tissue. This was treated with dressings until healing was complete at day 239.

### Local adverse reactions

Patient 2 exhibited signs of surrounding skin inflammation secondary to severe culture positive wound infection caused by contamination with infected urine. In patients 9 (Fig 4) and 10, where serous fluid collecting under the seal cultured positive for bacteria, the surrounding skin did not become inflamed, nor was inflammation seen around the wound containing the deep abscess in patient 8 (Fig 3). Patient 7 developed a reaction to the poliglecaprone sutures used to secure the graft first noticed on day 26 postgraft application, which settled after suture removal. No patients complained of pruritus or pain directly attributable to the BTM.

### Wound area

The ALT flap group (patients 1, 2, and 8) exhibited an overall slight increase in wound area during BTM integration, which then contracted to below the original size to around day 90 (representing a point of maximal scar activity). After this point the wound increased in size in both remaining patients over the remaining months of study.

The wound areas at grafting in 2 of the FOC flap group (patients 3 and 4) exceeded the original size. Patient 5's wound size (who was grafted at a later time point) initially increased to day 20 and then decreased over the next 9 days. Over the remaining months, all 3 wounds contracted to a mean of 69.15% of original size.

In the RF/UF flap group (patients 6, 7, 9, and 10), a decrease in wound area at skin grafting was noted in all patients (mean = 73.57%), which may reflect the overall trend of contraction relating to the increased time to graft afforded to these patients to allow for integration overexposed tendons (mean graft day = 38.5). These wounds contracted more than the other groups (power too small for statistical comparison), with a mean area of 36.64% of original size at 1 year.

### Scar outcomes

The POSAS Observer scale and MAPS scar assessments were completed on 8 participants at approximately 1 year (mean = 370.88 days) follow-up appointment. Only 7 participants were able to complete the POSAS Scale, as 1 patient (patient 5) was unable to communicate secondary to oral tumour recurrence. Table 2 contains a summary of scores from the 2 scar assessments. All participants reported no itch or pain (ie, score 1/10) from their scar in the previous 4 weeks on the POSAS Patient Scale.

## DISCUSSION

### Safety

In all cases, BTM was tolerated without symptom or sign of adverse reaction or hypersensitivity. Surrounding inflammation was attributable to either infection (patient 2) or reaction to poliglecaprone sutures (patient 7). No pain was reported in any donor site that was attributable to the BTM. Infection is addressed in a later section.

### Surgical experience

The BTM implantation was straightforward, and fixation rapid with staples. Overdressing was determined by our routine protocols for existing dermal templates.

### Integration and donor site variability

Rate of BTM integration differed between patients and donor sites. The determination that vascular integration is complete was subjective and based on our experience of the appearance of the matrix after delamination in successful porcine studies.^4^ Integrated BTM is adherent to its bed and displays a fine granulating superficial surface. The bonded seal is not designed to spontaneously delaminate once integration to the matrix surface has occurred. We had expected from these studies that the BTM would sustain skin grafting after 2 weeks. With patient 1, we learned that in humans this is not necessarily the case. In this case, the flap itself and the skin graft donor site healing were also delayed. Young, growing pigs with a strong immune system integrate BTM more rapidly than older, morbid, paraneoplastic human patients undergoing major and sometimes repeated surgical insult, enduring catabolic states and prolonged recovery times. We learned that longer times for integration are necessary in such patients.

The ALT and FOC donor sites appeared to integrate BTM at 3 to 4 weeks. The RF/UF donor sites required approximately 5 weeks to integrate over the volar wrist tendons.

The ALT donor site has a septum between rectus femoris and vastus lateralis opened to dissect free the perforators and pedicle of the flap. Such intra- and intermuscular dissection can traumatize and devascularize adjacent muscle. This open septum is sutured closed, prior to application of a split-skin graft routinely. Five days of bed rest allows skin graft to adhere to the bed; however, BTM appears to adhere more slowly. There may be a shear effect due to differential movement of the adjacent sutured muscles, inhibiting adherence of the BTM. Fluid can then collect underneath the BTM to form a small seroma (patient 1). We intervened in this situation with full-thickness BTM removal in this area. In patients 2 and 8 however, significant donor site complications secondary to underlying infection or necrotic muscle also required full-thickness removal of areas of BTM. We recommend careful debridement of any devitalized muscle at flap harvest, and closure of the septum over a drain.

The FOC donor site creates a deep cavity, which requires a drain and closure in layers. The BTM failed to adhere over the exposed distal peroneus longus tendon area in patient 3. This portion of the BTM was removed before grafting, leaving a defect over the tendon, which subsequently also failed to support overlying skin graft take. This was considered small enough to heal by secondary intention. This indicated that BTM integration over tendons takes longer, and further time should be allowed for this to occur, which modified our protocol for the later forearm flaps. In the authors’ experience, skin grafts alone in this site can fail and this can be prevented with a fascial-sparing approach. This was performed with success for patients 4 and 5.

The RF/UF donor sites have a convoluted base over tendons with preserved paratenon. These patients usually receive a forearm splint for 5 days to protect the skin graft from shear stress due to movement and this was increased to 7 days in this series. There was a tendency for tissue fluid to collect under the seal, encouraging blebs of delamination from 2 weeks postapplication. Secondary infection occurred in 2 such patients (9 and 10). The seal was windowed allowing fluid escape, resulting in control of the infection and preservation of the BTM through to integration. While skin grafts are routinely fenestrated in flap donor sites, the BTM seal is not (to prevent tissue ingrowth beyond the superficial surface of the BTM, which led to contraction in earlier unsealed matrices).^1^^-^4 In response to these findings, the BTM seal was hand-fenestrated for the second cohort (an ongoing trial).

### Infection

Any open wound and long-term synthetic implant carry with it a risk of infection, particularly if it remains exposed to the outside world via dressings. The BTM is no exception, although this risk has not yet been quantified. Localized infection was confirmed in 4 cases (patients 2, 8, 9, and 10). While 1 infection appeared attributable to muscle necrosis, the other 3 involved the BTM itself. Only patient 8 described considerable discomfort in the donor site overlying the abscess deep to the necrotic muscle. In this case, the BTM did not adhere over necrotic tissue and 16% was excised to debride muscle and drain the abscess. The wound responded to topical negative pressure therapy, and the remaining adjacent BTM integrated and was successfully skin grafted 1 week later. Patient 2 suffered a significant infection, which was treated nonoperatively with partial full-thickness removal of BTM (37%) and daily dressing changes until eradicated. In patients 9 and 10, partial removal of the seal alone allowed fluid escape and integration to continue without removal of any BTM. In all of these cases, the remaining BTM persisted to integration and ultimately sustained split-skin graft take.

### Delamination

The BTM seal was removed (delamination), by peeling with nontoothed forceps. The seal fragmented, requiring piecemeal removal in all patients. In a small wound, piecemeal delamination merely lengthened the process. However, this material is designed for use in major burn injury, where prolonged delamination might result in patient morbidity. This prompted immediate investigation into newer seals and seal-bonding methods in separate in vitro and in vivo animal studies. These improvements have already been tested in vivo in our porcine model, where rapid and single action delamination was observed.^7^ These new BTMs have been introduced in the human pilot burn trial and during their ongoing use in free flap donor site reconstruction.

### Skin grafting

Skin grafts were fixed over the integrated BTM with either suture or staples. The choice did not affect graft-take. Like other dermal templates, graft adherence is slightly delayed (compared to routine wound skin grafting) by 24 to 36 hours. We would recommend leaving the postsurgical dressing intact for 5 days, and delaying the commencement of physiotherapy, to accommodate this.

### Wound area

With a small number of patients, further separated into donor site groups, meaningful interpretation is difficult. Overall, the mean initial wound area decreases by 3.87% by the time of grafting (20-50 days). In our experience, this is less than wounds left to heal over these periods by secondary intention, supported by our research experience with control wounds in animals.^9^^,^^14^

The FOC and ALT donor sites initially increased in area. The wound bed muscles appeared to swell initially and then subside, and changes in measured wound area might be due to muscle volume changes altering the convexity of the limb and the measurement. After about 50 days in all 3 ALT patients, wound areas started to increase again, with the sole patient to survive to 12 months having a final wound area 118.6% of the original. Wound areas appeared to stabilize in the FOC patients after around day 80.

The RF/UF donor sites are flat or concave, with deeper grooves around the tendons. In these, the need for early, partial delamination in 3 patients facilitated granulation tissue formation and wound contraction.^4^ In all but the final patient (who suffered some graft loss over the tendon that healed by secondary intention and underwent more marked contraction), wound areas stabilized after around day 80.

These results are in keeping with our anecdotal experience of wound contraction, which tends to be at its most marked up to 90 days after split-skin grafting. With the exception of the ALT donor sites, even in an uneventful course, these data suggest that some wound contraction does occur, however without a larger scale randomized controlled trial comparison against an alternative dermal substitute, or immediate split-skin grafting, interpretation regarding resistance to contraction is not possible.

### Scar outcomes

Results for both the POSAS and MAPS scales favored the lower end of the scales indicating good scar characteristics and cosmetic outcome. A mean MAPS score of 1.88 in this study falls within mean scores of 1.5 (<10% grafting) and 2.4 (>10% grafting) previously reported by Jarrett et al^26^ 1 year after split-skin grafting of burn injuries. In a previous study, a mean Vancouver Scar Scale score of 4.2 was reported among 13 patients who underwent radial free flap donor site coverage with Integra and split-skin graft (mean follow-up of 23.8 months).^27^ Although comparison is difficult between scales, a mean MAPS score of 1.88 compares favorably. Mean follow-up time in this study was 370.88 days, indicating that there could also be more improvement as each scar matures and consequent further reduction in MAPS scores.

Mean POSAS total scores of 11.5 (Patient) and 15.75 (Observer) are lower than those reported for straight-line caesarean scars 1 year after wound closure.^28^ Average scores of 2.63 (Observer) and 1.88 (Patient) in the current study are also lower than those reported 12 months after full-thickness burn injury.^29^ These studies report on very different patient cohorts, with different etiologies and possibly different expectations.

Pain and itching have been reported to be a concern post–radial free flap donor site reconstruction. One study reported that 22% (patient group of 50) still had itching in their donor site over 6 months postprocedure,^30^ while another found 26% of patients with pain 1 year postoperatively following radial free forearm flap, albeit a low rating (0.5) on a visual analogue scale.^31^ Among the small sample in the present study, no patient reported itch or pain in their donor sites in the few weeks before undertaking the POSAS assessment.

### Polymer degradation and histopathology

**Figure 5:**
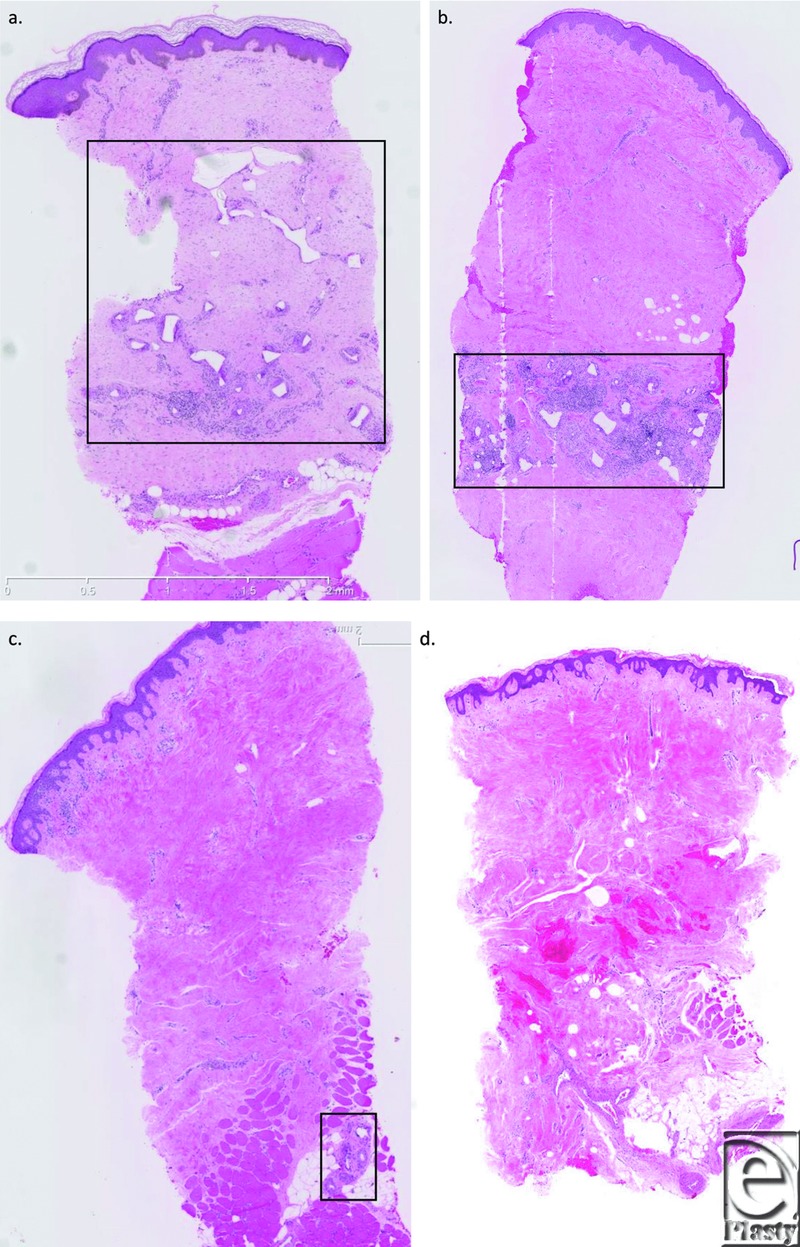
Punch biopsy sampling of integrated/overgrafted BTM. At 6 months (a), the 2-mm-thick material has undergone significant degradation and appears eroded with “rounded corners.” By 9 months (b), degradation has progressed, the polymer fragments are smaller and appear more—“spaced out” and rounded. At 12 months (c), degradation and absorption are almost complete with microscopic remnants remaining. The remnants (boxed) are no larger than the multinucleate macrophages surrounding them. By 18 months (d), there is no residual polymer.

The NovoSorb biodegradable polyurethane has been designed to maintain physical strength and structure until 3 months postapplication. After this time point, progressive hydrolysis of the material results in matrix degradation and absorption. This process is illustrated in Fig 5.

## CONCLUSIONS

This is an account of the first 10 patients to receive BTM as a dermal scaffold up to the point of 1 year or death due to unrelated events. Patients tolerate the polyurethane during this time and integration occurs after variable lengths of time, dependent on donor site and patient condition. Integrated BTM can sustain split-skin graft after its delamination and dermabrasion. Areas of nonintegrated BTM did not sustain split-skin graft, and further time to integration appears necessary over tendons. Subseal collections of fluid necessitated partial delamination of the seal to allow fluid escape (in 2 cases culture positive for bacteria). In 3 cases, partial removal of the full thickness of the BTM was necessary due to underlying muscle necrosis, wound infection, or seroma over which adherence and integration had not occurred. Remaining BTM can, however, subsequently persist and integrate to support skin graft-take. Wound contraction appears to stabilize after about 50 to 90 days; however, the degrees of this seen are benchmarks against which we have no comparison. Scars assessed after 1 year are favorable using both validated patient and observer related assessment tools.

## Figures and Tables

**Figure 2:**
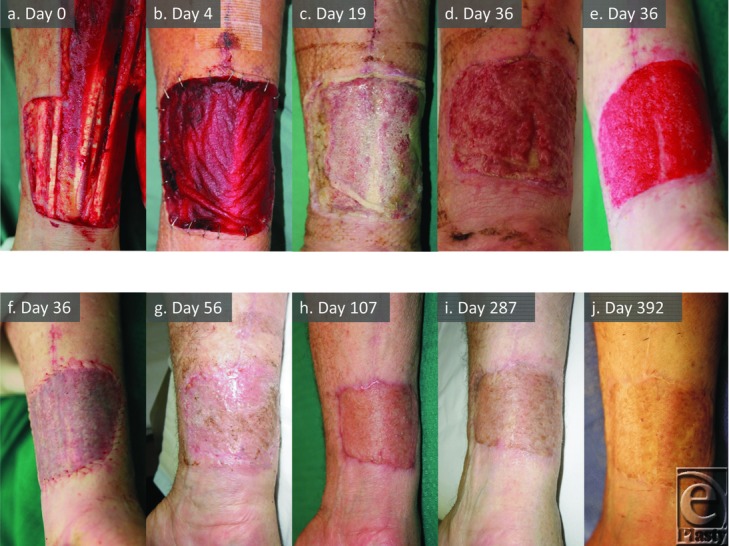
Patient 6 temporal series (UF). Demonstrates complex ulnar forearm free flap donor site (a), BTM integration (b-d), appearance post-dermabrasion (e), grafting (f), and graft maturation (g-j) to 1 year.

**Table 1:** Patient age, flap type, comorbidities, complications, BTM integration, and subsequent graft-take*^,^†

Pt	Age, y	Comorbidities	Flap	Complications	% BTM integration	Graft day	% wound area at grafting	Delamination	% take	% wound area at last reading, d
1	76	Aortic sclerosis, postural hypotension, paroxysmal atrial fibrillation, hypertension	ALT	Serous collection under BTM over intermuscular septum. Died of pneumonia	76	22 34	98.79 102.50	Piecemeal	20 100	91.73 (71)
2	73	Hypertension, type II diabetes mellitus	ALT	Infected donor site secondary to urinary tract infection requiring partial removal of matrix Died of metastasis	63	20	106.12	Piecemeal in the residual integrated BTM	100	127.54 (213)
3	72	Hypertension, ischaemic heart disease, CVA, sleep apnoea	FOC	BTM failed to integrate over peroneus longus tendon	90	20	119.89	Piecemeal	90	73.39 (363)
4	52	Nil	FOC	Nil	100	21	116.42	Piecemeal	100	66.57 (367)
5	52	Epilepsy, hypothyroidism, depression, alcoholic liver disease, previous subglottic SCC	FOC	Nil	100	29	90.92	Piecemeal	100	67.49 (363)
6	61	Angina, asthma, GORD	UF	Serous collection under BTM seal requiring early partial delamination	100	36	80.83	Piecemeal of residual seal	100	51 (392)
7	64	Hypertension, type II diabetes mellitus, ischaemic heart disease, COPD, gout	RF	Nil	100	34	78.37	Piecemeal	100	34.20 (367)
8	60	Hypothyroidism, gout	ALT	Necrotic rectus femoris muscle secondary to flap harvest, requiring partial removal of BTM at debridement	84	25	101.12	Piecemeal	100	118.60 (367)
9	46	Nil	RF	Infected collection under BTM seal requiring partial removal of seal	100	35	78.39	Piecemeal of residual seal	100	41.29 (365)
10	66	Hypertension, hypercholesterolaemia	RF	Infected collection under BTM seal requiring partial removal of seal	100	49	56.58	Piecemeal of residual seal	100	20.05 (383)

*ALT indicates anterolateral thigh flap; BTM, biodegradable temporizing matrix; COPD, chronic obstructive pulmonary disease; CVA, cerebrovascular accident or ‘stroke’ FOC, fibular osseocutaneous flap; GORD, gastro-oesophageal reflux disease; RF, radial forearm flap; SCC, squamous cell carcinoma; UF, ulnar forearm flap.

†Wound areas at the time of skin grafting and at final reading are expressed as percentages of the original wound area at the time of flap harvest.

**Table 2:** Scar assessment scores summary*

MAPS	
Average score	1.88 ± 1.25 (0-4)
POSAS	
Observer Scale	
Average score	2.63 ± 0.49
Overall opinion	2.63 ± 0.74 (2-4)
Patient scale	
Average score	1.88 ± 0.52
Overall opinion	2.29 ± 1.38 (1-4)

*Results are presented as mean ± SD (range).
